# Discriminative structural approaches for enzyme active-site prediction

**DOI:** 10.1186/1471-2105-12-S1-S49

**Published:** 2011-02-15

**Authors:** Tsuyoshi Kato, Nozomi Nagano

**Affiliations:** 1 Graduate school of Engineering, Gunma University, Tenjin-cho 1-5-1, Kiryu, Gunma, 376-8515, Japan; 2 National Institute of Advanced Industrial Science and Technology (AIST), Computational Biology Research Center, 2-42 Aomi, Koto-ku, Tokyo 135–0064, Japan; 3 Graduate School of Frontier Science, University of Tokyo, 5-1-5 Kashiwahoha, Kashiwa, Chiba 277-8561, Japan

## Abstract

**Background:**

Predicting enzyme active-sites in proteins is an important issue not only for protein sciences but also for a variety of practical applications such as drug design. Because enzyme reaction mechanisms are based on the local structures of enzyme active-sites, various template-based methods that compare local structures in proteins have been developed to date. In comparing such local sites, a simple measurement, RMSD, has been used so far.

**Results:**

This paper introduces new machine learning algorithms that refine the similarity/deviation for comparison of local structures. The similarity/deviation is applied to two types of applications, single template analysis and multiple template analysis. In the single template analysis, a single template is used as a query to search proteins for active sites, whereas a protein structure is examined as a query to discover the possible active-sites using a set of templates in the multiple template analysis.

**Conclusions:**

This paper experimentally illustrates that the machine learning algorithms effectively improve the similarity/deviation measurements for both the analyses.

## Background

The Enzyme Commission (EC) classification scheme for enzymes, which has been used worldwide for many years, is based mainly on the whole chemical structures of substrates and products, and on the cofactors involved [1]. However, because the EC classification scheme neglects protein sequence and structure information, it is sometimes difficult to detect a correlation between an enzyme sequence or structure and function based on EC classification. For instance, some homologous enzymes that are results of divergent evolution from the same ancestral enzyme might catalyze different reactions, whereas some nonhomologous enzymes from different super-families might catalyze the same reaction because of the convergent evolution. The enzyme pair of trypsin and subtilisin shares the Ser-His-Asp catalytic triad. It is a typical example of “analogous enzymes” produced by convergent evolution [2].

More recent reports suggest that such cases of active sites shared by analogous enzymes are not rare [3,4]. Considering those facts, Nagano developed an enzyme reaction database, EzCatDB, which provides a hierarchic classification of enzyme reactions, RLCP, which clusters the same reaction types together based on the reaction type, the reactive site of the substrate, the catalytic mechanism, and the catalytic site of enzymes [5]. Consequently, both the homologous reaction and the analogous reaction can be clustered together in the RLCP classification if they share the same catalytic mechanism and the catalytic site of the same type [5].

For enzyme-function prediction, particularly for detection of analogous enzymes, it is necessary to examine the specific local structures of the active sites that might reflect enzyme functions, rather than the global structures, such as the domain level or the chain level [6]. Regarding local structure comparison methods used to detect similar active sites, several “template-based” methods have been reported [7-15]. Those template-based methods search among target protein structures for the occurrence of a pre-defined template structure that consists of active-site residue atoms.

Our study specifically examines two practical applications of template-based methods. One is single template analysis, by which a single template is used as a query to search proteins for active sites that might have the same function as the template. Another is multiple template analysis, by which a particular protein structure is examined as a query to discover the possible active sites, whose structures resemble that in a set of templates. The key technique for both analyses is comparison of local site structures with template structures.

This paper presents machine learning techniques for two measurements to enhance the accuracy in comparing local sites with templates. One is an extension of the standard measurement: RMSD. The measurement parameterizes the deviation function so that the deviation can be refined by machine learning. Another measurement compares distance matrices instead of 3D structures. This measurement also employs machine learning to obtain a discriminative similarity between local sites and templates. In this work, the machine learning approaches are validated in the two applications: single template analysis and multiple template analysis. Our experimental results demonstrate the effectiveness of machine learning for both analyses.

**Figure 1:**
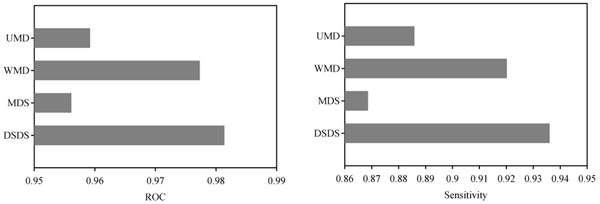
**Performances of prediction methods for single template analysis.** ROC is the area under the ROC curve. Sensitivity is computed at the specificity of 95%.

**Figure 2:**
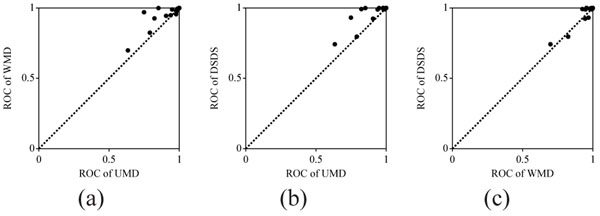
**Scatter plots of ROC scores in single template analysis.** (a) WMD vs. UMD; (b) DSDS vs. UMD; (c) DSDS vs. WMD. The scores for 36 templates are shown in each diagram.

**Figure 3:**
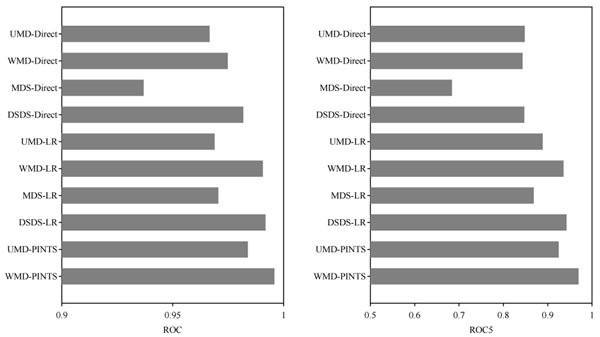
**Performances of prediction methods for multiple template analysis.** ROC is the area under the ROC curve. Sensitivity is computed at the specificity of 95%.

**Figure 4:**
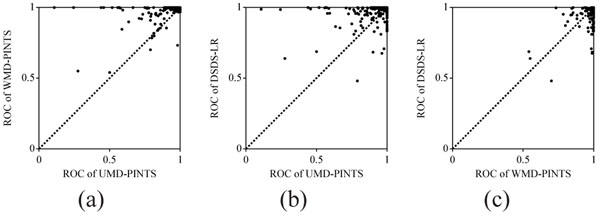
**Scatter plots of ROC scores in multiple template analysis.** (a) WMD-PINTS vs. UMD-PINTS; (b) DSDS-LR vs. UMD-PINTS; (c) DSDS-LR vs. WMD-PINTS. The scores for 1,219 protein structures are shown in each diagram.

Because of limited space, some figures and tables were not included in this paper. Figures 1, 2, 3, 4 and Tables 1, 2 are provided in the paper, although Figures 5–8 and Tables 3–5 are included in Supplemental Materials. The mathematical notations, the post-processing methods, and the abbreviations are also described in the Supplemental Materials.

**Table 1:** Example of search results obtained using the DSDS measurement in the single template analysis.

Rank	PDBid	Residues	Similarity
1	1ivr	TYR 217 A, LYS 250 A	2.282
2	1bkg	TYR 206 C, LYS 234 C	1.861
3	1arg	TYR 225 B, LYS 258 B	1.842
4	1bkg	TYR 206 A, LYS 234 A	1.829
5	1bkg	TYR 206 B, LYS 234 B	1.808
6	1arg	TYR 225 A, LYS 258 A	1.770
7	1bkg	TYR 206 D, LYS 234 D	1.769
8	5daa	TYR 31 B, LYS 145 B	1.683
9	1g4x	TYR 225 A, LYS 258 A	1.666
10	1oqu	TYR 266 A, LYS 270 A	1.656

Template 1ams is used to generate this example.

**Table 2:** Example of search results obtained using WMD-PINTS measurement in multiple template analysis.

	Residues	Template	log(*F*_pints_)	Deviation
1	HIS 57 A, ASP 102 A, GLY 193 A, SER 195 A	1acb	–53.37	0.83
2	ASP 137 A, ASP 194 A	1qk2	–14.00	0.66
3	ASP 137 A, ASP 194 A	2bvw	–12.86	0.69
4	ASP 100 A, ASP 97 A	1qk2	–7.77	0.85
5	ASP 189 A, HIS 172 A	1emh	–6.60	1.10
6	ASP 102 A, ASP 97 A	1qk2	–5.18	0.95
7	ASP 239 A, ASP 128 A	1qk2	–5.11	0.95
8	ASP 194 A, ASP 189 A	2bvw	–5.08	0.96
9	ASP 97 A, HIS 57 A	1emh	–2.79	1.25
10	ASP 61 A, HIS 40 A	1emh	–2.62	1.26

Protein structure 1bio is used to generate this example.

## Results and discussion

### Overview of experiments

This section presents experimental results to underscore the performance of template-based active-site prediction algorithms. Template is definable as a set of atoms in an active site of an enzyme protein. Roughly speaking, active-site prediction is performed by comparing the active sites of templates with local sites in protein structures; if the local site structure is sufficiently similar to the active-site template, the local site can be predicted as an active site. Precisely, there are two typical ways of predicting active sites using templates: *single template analysis* and *multiple template analysis*.

#### Single template analysis

In the single template analysis, it was attempted to search protein structures for local sites similar to a query template, to discover proteins that have the same active site as the template. The input and output in the single template analysis are described as follows:

**Input (query):** Template structure.

**Output:** List of proteins in a protein structure database. The proteins in the list are attached with the coordinates of local sites that are similar to the query template.

An example of the output in the single template analysis is given in Table 1. Template 1ams is used to generate the list. The top 10 proteins are listed as sorted in descending order of the similarity values. The predicted local sites in the 10 proteins are also included in the list. Indeed, all the 10 proteins have the same function as that of the template, and their active sites coincide exactly with the listed sites.

A typical procedure of single template analysis consists of two stages as follows:

1. **Local site search.** A local site search (LSS) algorithm, such as TESS [7] and JESS [11], is used to enumerate the candidates of local site structures similar to the template.

2. **Similarity/deviation computation.** The similarity or deviation of each candidate site to or from the template is computed. The candidate sites are sorted in descending and ascending order of the similarity and deviation values.

#### Multiple template analysis

Multiple template analysis is attempted to find active sites in a query protein structure by searching for local sites that are similar to a template structure in a set of pre-defined templates. This is used for predicting the function of the query protein.

**Input (query):** Protein tertiary structure.

**Output:** List of local sites that are similar to a pre-defined template.

The list in Table 2 is an example of the output in the multiple template analysis. This result is generated by attempting to predict the active sites in a query protein structure of 1bio. The list contains 10 local sites, which are sorted according to the confidence level. The true active-site in 1bio is “HIS 57 A, ASP 102 A, GLY 193 A, SER 195 A”, which is predicted at the top of the list.

A typical procedure of multiple template analysis consists of three stages:

1. **Local site search.** A local site search (LSS) algorithm is used to enumerate the candidates of local structures similar to a template.

2. **Similarity/deviation computation.** The similarity or deviation of each candidate site to or from the corresponding template is computed.

3. **Post-processing.** The similarities or deviations above are transformed to some probabilistic scores to compare and sort the local sites in descending order of the score values.

### Experimental settings

To investigate the performance of the templates, a search experiment was conducted against the PDB protein structure dataset. An enzyme database, EzCatDB [5], which contains information related to catalytic reaction classification and active-sites of enzymes, was used for the experiment. EzCatDB provides a hierarchic classification of enzyme reactions, RLCP, which clusters the same reaction types together based on the reaction type, the reactive site of the substrate, the catalytic mechanism, and the active-site of enzymes [5]. The RLCP classification differs from the conventional enzyme classification, EC, as described in the Background section. In all, 36 templates were prepared based on the RLCP classification. To evaluate the prediction methods, protein structures that are assigned to have the same RLCP class as one of the 36 templates were enumerated for use in our experiments. Consequently, 1,219 structures were obtained.

A template can be created from a set of amino acid residues in an active-site from an EzCatDB entry. In the EzCatDB database, each residue in the active-site is classified into one of four types: catalytic-site residue, cofactor-binding-site residue, modified residue, and mainchain catalytic residue. For catalytic-site residues and modified residues, atoms from the sidechains of residues are included automatically in the template, whereas all atoms are included in the template for cofactor-binding-site residues. For mainchain catalytic residues, only the mainchain atoms are included in the template. The 36 templates were created by this procedure. In Table 3 (suppl.), the original PDBids for those templates are listed.

In our study, TESS [7] was implemented as an LSS algorithm to perform the local site search that is used for the first step in the catalytic-function prediction system. The LSS algorithm was applied to the 1,219 protein structures, and local sites that have RMSD larger than 4.0 Å were removed from the detected local structures. As a result, 587,431 local structures were detected and used for our experiments. Then, local sites were labeled as positive sites for a template if they were annotated in EzCatDB to have the same function as the template; the other local sites were labeled as negative sites. Some templates hit many local sites, but others hit only a few. Template 1acb hits the largest number of positive sites among 36 templates, and the number is 557. The template 1qk2 detected 108,036 negative sites, which is the greatest number among the 36 templates. The medians of the quantities of positive and negative sites are, respectively, 42 and 3401.5.

Mostly, only a few positive sites were detected in each protein structure by the LSS algorithm, although many more negative sites were detected. The distribution of the detected sites in the multiple template analysis is shown in the histograms (Figure 5 (suppl.)), where the x-axis shows the number of detected sites for each query protein and the y-axis shows the frequency of hit proteins. Therein, the same local sites can be detected several times when the sites are detected by several different templates. Only one positive site was detected among 849 protein structures. The remaining 370 protein structures have multiple positive sites. About 95.5% of protein structures contain fewer than five positive sites. The number of negatives is much greater than the number of positives in most protein structures. The median of the number of negative sites in a protein structure is 52. The 95th percentile is 163, and the maximum number is 720. This fact motivated us to devise precise similarity or deviation measurements between local site structures and template structures in order to extract true positive sites among the vastly numerous local sites detected in a protein structure.

To examine the generalization performance of prediction algorithms, the dataset of 1,219 PDB entries was divided randomly into a training set and a test set, so that each dataset can have 50% of the original dataset. The divisions were then adjusted so that at least one active-site can be hit in a training set by the LSS algorithm for every template. The test set was used only for prediction. Consequently, the test PDB set was never used for learning. This procedure was repeated 30 times. The average of the prediction performances over the 30 trials is described in this section.

### Single template analysis

To predict local sites detected by the LSS algorithms, precise similarity or deviation measurements are necessary. Currently, the standard measurement is the so-called RMSD. Our study introduces two measurements: *Weighted Mean Deviation* (WMD) and *DALI Score-based Discriminative Similarity* (DSDS). RMSD is computed by taking the unweighted average of square Euclidean distances, whereas WMD takes the weighted average of distances. Furthermore, DSDS is the linear combination of DALI scores. The parameters for both WMD and DSDS are obtained using machine learning algorithms, which will hereinafter show notable differences from the conventional measurements in prediction performance. To confirm the effectiveness of machine learning for DSDS, the *mean of DALI scores* (MDS) was tested as the similarity between local sites and templates for the experimental control. The square of RMSD is designated as the Unweighted Mean Deviation (UMD), revealing the same ranking as RMSD.

An example of the prediction results in the single template analysis is shown in Table 1. This is a result obtained by the DSDS measurement, using template 1ams. All local sites in the list are positive, as described above. For comparison, Table 4 (suppl.) portrays a prediction result obtained using the UMD measurement, with the same template. Unfortunately, all the local sites in the list are negative.

To evaluate the performance of active-site prediction algorithms for the single template analysis, two criteria were adopted: ROC score and Sensitivity. The ROC score is the area under the ROC curve that is shown in two-dimensional space where the x-axis shows the false positive rate (FPR), and the y-axis shows the true positive rate (TPR). As a discrimination threshold of the similarity/deviation varies, different FPRs and TPRs are obtainable, yielding many points in the FPR-vs.-TPR space. Connecting those points yields an ROC curve for each template and for each of 30 trials. Here, the average of ROC scores was obtained. Sensitivity is also evaluated where the discrimination threshold is adjusted so that the specificity is 95%. In this article, the capitalized word, Sensitivity, was adopted to denote the sensitivity at specificity of 95%.

Figure 1 depicts the average of ROC scores and Sensitivities over the 36 templates, respectively. Comparing the four similarity or deviation measurements, DSDS achieved the best ROC score and the best Sensitivity (ROC score of 0.981 and Sensitivity of 0.936). WMD obtained the ROC score of 0.977 and Sensitivity of 0.920, each of which is the second best among the four similarity or deviation measurements. The difference between DSDS and WMD is small; one-sample *t*-test was insufficient to detect the statistically significant changes in both ROC score and Sensitivity (*P*-values are respectively 0.106 and 0.181 ). Compared to UMD, WMD obtained a significant improvement; the changes in ROC score and Sensitivity are 0.0181 and 0.0343, respectively, and the *P*-values of the changes are 0.00495 and 0.00356, respectively. The improvements from MDS to DSDS were larger. The changes in ROC score and Sensitivity are respectively 0.0253 and 0.0674. The results suggest that the combination of the DALI-score with machine learning is more effective than that of mean deviation.

Figure 2 and Figure 6 (suppl.) show scatter plots of the ROC scores and the Sensitivities, respectively, to compare three measurements—UMD, WMD, and DSDS—in template-by-template fashion. The two measurements obtained using machine learning, WMD and DSDS, performed much better than the baseline measurement: UMD. No remarkable difference between WMD and DSDS was observed from the scatter plots.

Figure 7 (suppl.) plots the median ROC curves over 36 templates. The median ROC curve is obtained by computing an ROC curve for each template, and taking the median over 36 TPRs at every FPR. To investigate the change in the ROC curves from different templates, the curves of the 25th percentiles and the 75th percentiles are also shown. The median ROC curve is drawn by solid curves, and the 25th percentiles are shown as dotted curves under the median curve, and the 75th percentiles are by dotted curves over the median curve. The changes in the ROC curves from different templates are not small for every prediction method. Another notable point is that the TPR value on the 25th percentile for DSDS is markedly higher than that for MDS; MDS and DSDS yield TPR values of 0.823 and 0.966 at the 5% FPR on the 25th percentiles, respectively, which implies that DSDS performs stably compared to MDS.

### Multiple template analysis

To perform multiple template analysis for predicting the function of a query protein structure, the similarity or deviation measurements, such as RMSD, must be transformed through post-processing into some unified scores, which can be compared among different templates. One post-processing is the *logistic regression* (LR) [16]. The LR method provides posterior probabilities for given similarity or deviation values. The parameters of the posterior probability function are estimated using training data. Another post-processing is *PINTS*[17]. Results of our experiments revealed that PINTS works well for the square root of WMD. However, PINTS can be applied to neither MDS nor DSDS.

Methods of experimental control, designated as *Direct* in this paper, which compare the similarity or deviation measurements directly, were also tested. In the Direct methods, post-processing is not conducted. Consequently, there are now 10 combinations of the similarity or deviation measurements using post-processing methods: UMD-Direct, WMD-Direct, MDS-Direct, DSDS-Direct, UMD-LR, WMD-LR, MDS-LR, DSDS-LR, UMD-PINTS, WMD-PINTS.

An example presented in Table 2 was generated using WMD-PINTS. The local sites in the list are the predicted results for active-sites in the protein structure 1bio. As described above, the top in the prediction results is the true positive site. Table 5 (suppl.) portrays a prediction result obtained using the UMD-PINTS measurement, to predict the active sites of the same protein structure 1bio. In this case, no active site was predicted in the top 10.

To evaluate the prediction performance in the multiple template analysis, an ROC curve is drawn for each protein structure to compute the area under the curve: the ROC score. A modified version of the ROC score, called ROC5 score [18], is also used for performance evaluation. The ROC5 score is the area under ROC curve up to the first five false positives; the score is scaled so that it will be 0–1.

Figure 3 presents the average ROC score and the average ROC5 score, respectively, across 1,219 proteins for each measurement. Logistic regression engenders improvements in all sensitivity/deviation measurements, and PINTS further improves the performance. The WMD-PINTS achieved the ROC of 0.996 and the ROC5 of 0.970, which are significantly better than any of the four measurements using logistic regression. The best measurement among those four measurements using logistic regression is DSDS-LR, which yields the ROC of 0.991 and the ROC5 of 0.942. Actually, DSDS-Direct obtained the best ROC among the methods without post-processing (ROC of 0.982) and UMD-Direct obtained the best ROC5 among those four methods (ROC5 of 0.848), although they are significantly worse than either WMD-PINTS or DSDS-LR.

To compare the machine learning-based methods with the experimental control, the ROC and ROC5 scores of the three methods, UMD-PINTS, WMD-PINTS, and DSDS-LR are shown in the scatter plots of Figure 4 and Figure 8 (suppl.). These three are, respectively, the best methods among the methods with UMD, WMD, and DSDS. Figure 4 shows the ROC scores for 1,219 PDB structures, and Figure 8 (suppl.) portrays the ROC5 scores. For both the criteria, the two methods, WMD-PINTS and DSDS-LR, performed much better than the baseline method, UMD-PINTS, which suggests that machine learning is effective for the multiple template analysis. No remarkable difference between the two machine-learning-based methods were observed.

## Conclusions

This paper presented an examination of the effectiveness of novel machine-learning-based similarity or deviation measurements for comparison of template structures with local site structures in proteins. The measurements are applicable to two situations: one for seeking proteins that have a particular catalytic function by comparing a template with local sites in those proteins (single template analysis), and another for predicting the function of a particular protein by comparing local sites in the protein structure with a set of templates whose functions are annotated (multiple template analysis). Our experimental results demonstrated that, for both situations, machine learning-based methods performed better than the conventional methods. Further improvements in the machine learning methods presented in this paper by transferring information from the learning of other templates [19,20] or by incorporation of additional information such as sequence and ligand structure into our current methods are anticipated as interesting avenues for future work.

## Methods

### Similarity/deviation computation

Both the single template analysis and the multiple template analysis measure how similar the local site structures are to the template structures, or how large the deviations of the local sites are from the template structures. It would be favorable if the active sites with the same catalytic functions as the template obtained higher ranks than the other local sites. This study introduced four similarity/deviation measurements: UMD, WMD, MDS, and DSDS.

#### Unweighted mean deviation (UMD)

Conventionally, the root of unweighted mean square deviation, also known as RMSD, has been used to measure the deviation of local sites from the corresponding templates. This paper refers to the square of RMSD as UMD, and reviews it here. Presuming that a template *T_k_* consists of *m_k_* atoms, and that a local site *S_j_* that was searched with the template *T_k_* contains *m_k_* atoms, then each atom in the local site *S_j_* has one-to-one correspondence with one of atoms in the template. The 3D coordinate of the *i*-th atom in the template *T_k_* is denoted by ***t****_i_*_,_*_k_* ∈ ℝ^3^. The 3D coordinate of the *i*-th atom in *S_j_* is denoted by ***s****_i,j_* ∈ ℝ^3^. The value of UMD of *S_j_* from *T_k_* is given as

where **O** is the set of rotation matrices. Consequently, the deviation is measured using the optimal rigid-body transformation (***A****_j_*, ***b****_j_*) [21].

#### Weighted mean deviation (WMD)

A generalization of UMD, WMD is introduced here. The importance of each atom in a template is heterogeneous. Some atoms play an important role in catalytic reaction, and others do not. The positions of some atoms are well conserved; those of others are not. Even if the positions are not strongly conserved, those might be conserved to a greater degree than the atoms of non-active sites. To describe the importance of atoms, the importance parameters  are introduced. The values are constrained to be non-negative, and the sum of *m_k_* values is one. Using the importance parameters, WMD is defined as

The values of the importance parameters are determined using a machine learning algorithm described later. An important issue of WMD against the importance parameters arises from the superimposition. The fact that the optimal superimposition depends on the values of the importance parameters complicates the learning algorithm. The measurements shown in the next paragraphs are defined without superimposition to avoid such an issue.

#### Mean DALI score (MDS)

DALI is well-known software which performs alignment of protein structures [22]. The algorithm aligns the distance matrices among protein residues; thereby superimposition is avoided during alignment [22]. The measurement MDS adopts the score function of DALI, which measures how similar an atom pair in one structure is to an atom pair in the other structure. Given an atom pair (*i*_1_, *i*_2_) in a template and the corresponding local site, the DALI score function is defined as

where  is the Euclidean distance between the *i*_1_-th atom and the *i*_2_-th atom in the template, and where  is the Euclidean distance between the *i*_1_-th atom and the *i*_2_-th atom in the local site. In addition,  is the average of  and . In fact, MDS is the mean of the DALI scores for *m_k_*(*m_k_* – 1)*/*2 atom pairs.

#### DALI score-based discriminative similarity (DSDS)

We have already argued that some atoms are relevant to prediction, and that others are not. Nevertheless, the MDS measurement deals with all the atom pairs equally. For use in this paper, DSDS is defined as the linear combination of the DALI scores,  with coefficients, :

The coefficients ***w****_k_* are determined using a machine-learning algorithm, which is presented following the description of the learning algorithm for WMD.

### Learning algorithms

Both the importance parameters of WMD, ***β****_k_*, and the coefficients of DSDS, ***w****_k_*, are determined using a training dataset. Training datasets are created by application of the LSS algorithm to a set of annotated protein structures. In the dataset, local sites are labeled as positive sites if they are annotated to have the same function as the template; the other local sites are labeled as negative sites.

#### Learning algorithm for WMD

The importance parameters included in WMD, ***β****_k_*, are determined as a learning algorithm described here. It is favorable to obtain the importance parameters ***β****_k_* that yield small deviations for positive sites, and large deviations for negative sites. In this work, a loss function  was designed to represent the quality of the importance parameters. A discrimination threshold *θ_k_* and a small non-negative constant *ε* are considered here. For a positive site, the function  gives the absolute difference between the threshold *θ_k_* – *ε* and the deviation *δ_j_* if the deviation is greater than *θ_k_* – *ε*. For a negative site, the difference between the threshold *θ_k_* + *ε* and the deviation *δ_j_* is measured if the deviation is less than *θ_k_* + *ε.* No loss will be given for a positive (negative) site if the deviation of the local site is less (greater) than *θ_k_* – *ε* (*θ_k_* + *ε*). Mathematically the loss function is expressed as

where *y_j_* is +1 if *S_j_* is a positive site, and –1 otherwise. Using pre-determined weights *C_j_* for local sites, the learning algorithm is used to minimize the sum of the losses

for all training data with respect to the importance parameters ***β****_k_* and the threshold *θ_k_*. Therein, ***H****_k_* is the index set of local sites corresponding to template *T_k_*. However, the formulation is reduced to a bi-level programming problem [23] because the definition of the deviation already includes a minimization problem with respect to rigid-body transformation. In general, bi-level optimization is not tractable to find the global minimizer. In our work, a two-stage iterative algorithm was used to solve the problem. The first stage optimizes the importance parameters, fixing the rigid-body transformations. The second stage optimizes rigid-body transformation for each local site, fixing the importance parameters. The two stages are repeated several times. The sub-problem for the first stage is reduced to a linear program. The GNU Linear Programming Kit (http://www.gnu.org/software/glpk/) was adopted to solve the linear program. The sub-problem for the second stage is solvable by a slight modification of Procrustes analysis [24]. Although the iterative algorithm is not theoretically guaranteed to perform global optimization, we found in the experiments that the algorithm works well.

In our experiments, the number of iterations for the iterative algorithm was set to 2. The loss of each local site was weighted by *C_j_* and summed up to form the total loss. The weights for positive sites are set to 1*/n_k_*_,+_, and the weights for negative sites are set to 1*/n_k_*_,–_ where *n_k_*_,+_ and *n_k_*_,–_ respectively denote the number of positive sites and negative sites by template. To avoid over-fitting, we give an upper bound 2*/m_k_* to the importance parameters.

#### Learning algorithm for DSDS

To determine the values of the coefficients ***w****_k_*, the learning algorithm of the support vector classifier (SVC) algorithm [16] can be used. SVC attempts to find the values of the coefficients ***w****_k_*, so that the similarities of all positive sites cannot be smaller than *θ_k_* + *ε*, and so that the similarity scores of all negative sites cannot be greater than *θ_k_* – *ε*. Similar to the learning algorithm for WMD, the violations of the conditions are expressed in the loss function as

Typically, *ε* is set to 1. To avoid over-fitting of the prediction model to the training dataset, the SVC learning algorithm adds a regularization term to the objective function, engendering the following objective function:

The SVC learning algorithm efficiently minimizes the objective function with respect to the coefficients ***w****_k_* and the discrimination threshold *θ_k_*. Our experiments used an implementation of the SVC learning algorithm, LIBSVM (http://www.csie.ntu.edu.tw/~cjlin/libsvm), to obtain the values of the coefficients. The constants *C_j_* are set to 1,000*/n_k_*_,+_ for positive sites, and to 1,000*/n_k_*_,–_ for negative sites.

## Authors' contributions

TK designed and implemented the algorithms, performed the experiments, and drafted the manuscript. NN provided the data and revised the manuscript. All authors read and approved the final manuscript.

## Competing interests

The authors declare that they have no competing interests.

## Supplementary Material

Additional file 1 **Supplementary Materials** This file provides the mathematical notations, the post-processing methods, the abbreviations, Figure 5–8, and Tables 3–5. Click here for file

## References

[B1] Webb EC Enzyme Nomenclature 1992. Recommendations of the Nomenclature Committee of the International Union of Biochemistry and Molecular Biology 1992 New York: Academic Press Inc

[B2] Wright CS Comparison of the active site stereochemistry and substrate conformation in chymotrypsin and subtilisin BPN’ J Mol Biol 1972 67 151 63 10.1016/0022-2836(72)90391-9 5064999

[B3] Gherardini PF Wass MN Helmer-Citterich M Sternberg MJ Convergent evolution of enzyme active sites is not a rare phenomenon J Mol Biol 2007 372 3 817 45 10.1016/j.jmb.2007.06.017 17681532

[B4] Nagano N Noguchi T Akiyama Y Systematic comparison of catalytic mechanisms of hydrolysis and transfer Proteins 2007 66 147 59 10.1002/prot.21193 17039546

[B5] Nagano N EzCatDB: the Enzyme Catalyticmechanism Database Nucleic Acids Res 2005 33 Database issue D407 12 10.1093/nar/gki080 15608227 PMC540034

[B6] Loewenstein Y Raimondo D Redfern OC Watson J Frishman D Linial M Orengo C Thornton J Tramontano A Protein function annotation by homology-based inference Genome Biol 2009 10 2 207 10.1186/gb-2009-10-2-207 19226439 PMC2688287

[B7] Wallace AC Borkakoti N Thornton JM TESS: a geometric hashing algorithm for deriving 3D coordinate templates for searching structural databases. Application to enzyme active sites Protein Sci 1997 6 11 2308 2323 10.1002/pro.5560061104 9385633 PMC2143595

[B8] Fetrow JS Skolnick J Method for prediction of protein function from sequence using the sequence-to-structure-to-function paradigm with application to glutaredoxins/thioredoxins and T1 ribonucleases J Mol Biol 1998 281 5 949 68 10.1006/jmbi.1998.1993 9719646

[B9] Kleywegt GJ Recognition of spatial motifs in protein structures J Mol Biol 1999 285 4 1887 97 10.1006/jmbi.1998.2393 9917419

[B10] Stark A Russell RB Annotation in three dimensions. PINTS: Patterns in Non-homologous Tertiary Structures Nucleic Acids Res 2003 31 13 3341 4 10.1093/nar/gkg506 12824322 PMC168913

[B11] Barker JA Thornton JM An algorithm for constraint-based structural template matching: application to 3D templates with statistical analysis Bioinformatics 2003 19 13 1644 9 10.1093/bioinformatics/btg226 12967960

[B12] Chou KC Cai YD A novel approach to predict active sites of enzyme molecules Proteins 2004 55 77 82 10.1002/prot.10622 14997541

[B13] Ivanisenko VA Pintus SS Grigorovich DA Kolchanov NA PDBSiteScan: a program for searching for active, binding and posttranslational modification sites in the 3D structures of proteins Nucleic Acids Res 2004 32 Web Server issue W549 54 10.1093/nar/gkh439 15215447 PMC441577

[B14] Laskowski RA Watson JD Thornton JM Protein function prediction using local 3D templates J Mol Biol 2005 351 3 614 26 10.1016/j.jmb.2005.05.067 16019027

[B15] Torrance JW Bartlett GJ Porter CT Thornton JM Using a library of structural templates to recognise catalytic sites and explore their evolution in homologous families J Mol Biol 2005 347 3 565 81 10.1016/j.jmb.2005.01.044 15755451

[B16] Hastie T Tibshirani R Friedman JH The Elements of Statistical Learning 2003 Springer

[B17] Stark A Sunyaev S Russell RB A model for statistical significance of local similarities in structure J Mol Biol 2003 326 5 1307 16 10.1016/S0022-2836(03)00045-7 12595245

[B18] Gribskov M Robinson NL Use of receiver operating characteristic (ROC) analysis to evaluate sequence matching Comput Chem 1996 20 25 33 10.1016/S0097-8485(96)80004-0 16718863

[B19] Kato T Kashima H Sugiyama M Asai K Platt J, Koller D, Singer Y, Roweis S Multi-Task Learning via Conic Programming Advances in Neural Information Processing Systems 20 2008 Cambridge, MA: MIT Press 737 744

[B20] Kato T Okada K Kashima H Sugiyama M A Transfer Learning Approach and Selective Integration of Multiple Types of Assays for Biological Network Inference International Journal of Knowledge Discovery in Bioinformatics (IJKDB) 2010 1 66 80

[B21] Kato T Tsuda K Tomii K Asai K A new variational framework for rigid-body alignment Structural Syntactic, and Statistical Pattern Recognition 2004 3138 Springer Berlin / Heidelberg 171 179 full_text

[B22] Holm L Sander C Protein structure comparison by alignment of distance matrices J Mol Biol 1993 233 123 38 10.1006/jmbi.1993.1489 8377180

[B23] Colson B Marcotte P Savard G An overview of bilevel optimization Annals of Operations Research 2007 153 235 256 10.1007/s10479-007-0176-2

[B24] Kendall DG A Survey of the Statistical Theory of Shape Statistical Science 1989 4 2 87 120 10.1214/ss/1177012582

