# Supplementary material for: Discriminative structural approaches for enzyme active-site prediction
Source: BMC Bioinformatics. 2011 Feb 15;12(Suppl 1):S49. doi: 10.1186/1471-2105-12-S1-S49 (PMC3044306; doi:10.1186/1471-2105-12-S1-S49)
Supplement: Additional file 1 — Supplementary Materials This file provides the mathematical notations, the post-processing methods, the abbreviations, Figure 5–8, and Tables 3–5. [file 1471-2105-12-S1-S49-S1.pdf]

Supplemental Materials: Discriminative Structural Approaches for Enzyme Active-Site Prediction

Tsuyoshi Kato and Nozomi Nagano

August 15, 2010

Notations

Vectors are denoted by boldface lower-case letters and matrices, by boldface upper-case letters. Elements of vectors and matrices are printed in lightface. The transposition of matrix $\boldsymbol{A}$ is denoted by $\boldsymbol{A}^\top$. $\mathbb{R}$ is used to denote the set of real numbers, $\mathbb{R}^n$ to denote the set of n-dimensional real column vectors. The set of real nonnegative numbers is denoted by $\mathbb{R}_+$. $\mathbb{N}_n$ is a set of natural number less than or equal to n. Symbols $\leq$ and $\geq$ are used to denote not only the standard inequalities between scalars, but also the componentwise inequalities between vectors. For $\forall x \in \mathbb{R}$, the operator $[x]_+$ returns x if $x \geq 0$; otherwise, it returns 0.

Post-Processing

Logistic Regression

In the multiple template analysis, the similarities have to be compared between different templates. As described above, the DSDS parameters are adjusted, so that the scores for positive sites can be positive and those for negative sites can be negative. The importance parameters for WMD can be adjusted automatically, so that a threshold can separate the deviations of positive sites from those of negative sites. However, the similarities or the deviations still cannot be comparable between different templates. This is because each template has unique threshold value for WMD, and the magnitudes of the DSDS scores are various among the templates.

The logistic regression is employed in this work, so that the results by different templates can be compared with each other. Given the similarity or deviation values, the logistic regression model provides the posterior probabilities that the local site S_j can be the same active-site as a corresponding template T_k. For an unknown site with deviation δ_{unk}, the posterior probability of being an active site (i.e. $y_{\text{unk}} = +1$) is given by

$$P(y_{\text{unk}} = +1 \,|\, \delta_{\text{unk}}, T_k) = \frac{e^{a_{1,k}\delta_{\text{unk}} + a_{2,k}}}{1 + e^{a_{1,k}\delta_{\text{unk}} + a_{2,k}}}$$

where $(a_{1,k}, a_{2,k})$ is the parameter set of the logistic regression model. Penalized maximum likelihood estimation is performed to determine the values of the parameters $(a_{1,k}, a_{2,k})$.

PINTS

PINTS [1] is a probabilistic model that enables us to convert incomparable RMSDs into comparable P-values. The probabilistic model provides a look at how likely the specified RMSD can happen by chance, to allow us to detect the statistical significance of the RMSD from local sites to templates. The P-values are given by $1 - \exp(-F_{\text{pints}})$, where F_{pints} is the expected number of matches with a certain RMSD, which is smaller than the observed RMSD. The formula of F_{pints} includes many parameters that are adjusted empirically by observation of real world protein structures and the values of RMSD [1]. Although WMD is not the standard RMSD, the P-values were adopted using the parameter values from the literature [2] without adjustment in our work. Nevertheless, the P-value works very well in our experiments, which will be described in the section of experimental results. For MDS or DSDS, PINTS cannot be employed.

Abbreviations

- LSS : Local Site Search
- UMD : Unweighted Mean Deviation
- WMD : Weighted Mean Deviation
- MDS : Mean DALI Score
- DSDS : DALI Score-based Discriminative Similarity
- LR : Logistic Regression.
- RMSD : Root Mean Square Deviation
- TPR : True Positive Rate
- FPR : False Positive Rate
- ROC : Receiver Operating Characteristics

Table 3: Templates used in our experiments

RLCP Classification	CATH Classification	PDBid
1.12.30000.14	3.40.50.1820	2ace
1.13.11100.261	3.40.390.10	1af0
1.13.11110.262	3.40.630.10	3cpa
1.13.200.966	2.40.70.10	1psa
1.13.30000.10	2.40.10.10	1acb
1.13.30000.16	3.40.710.10	1bls
1.15.10100.1172	1.10.473.10; 3.30.70.370; 3.30.420.10	1kfs
1.15.30200.84	3.40.50.1240	1rpa
1.15.60000.82	3.90.730.10	1vcz
1.15.7910.1164	3.30.540.10; 3.40.190.80	1ka1
1.15.8230.362	2.70.40.10	2oke
1.15.9400.1180	3.40.600.10	1eo4
1.20.30810.950	3.40.50.1820	2dhc
1.20.30810.951	3.40.50.1820	1g42
1.30.260.1001	2.160.20.10	1kcd
1.30.300.2	3.20.20.40	1qk2, 2bvw
1.30.36000.3	3.20.20.80	1bg9, 1jfh
1.30.36010.970	3.20.20.80	1isw
1.40.4930.61	3.40.470.10	1emh
3.103.130000.1162	1.10.510.10	1gjo
3.113.90000.397	1.10.1160.10; 3.40.50.620	1euq
3.133.90010.394	3.40.50.300	1zio
5.1202.1504200.6501	3.20.20.70	1e51
6.10.398000.111	3.20.20.70	1h7p
6.10.82600.5900	3.40.640.10	1ams
6.20.85200.5520	3.40.640.10	1ams, 1map
6.30.97700.5320	3.40.640.10	1ahy, 1arg
6.40.521000.5530	3.40.640.10	1ahg
8.113.42001.5	3.20.20.70	4tim
8.121.1440000.6450	3.20.20.70	1eb3
8.131.42001.6	3.20.20.70	6tim
8.311.591510.5526	3.40.640.10	1arg
8.311.591510.5527	3.40.640.10	1cq7

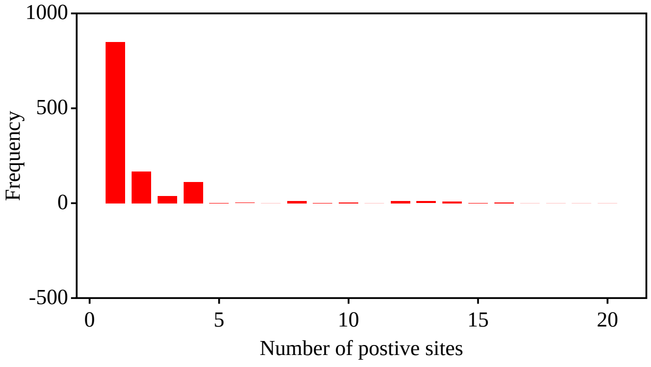

(a)

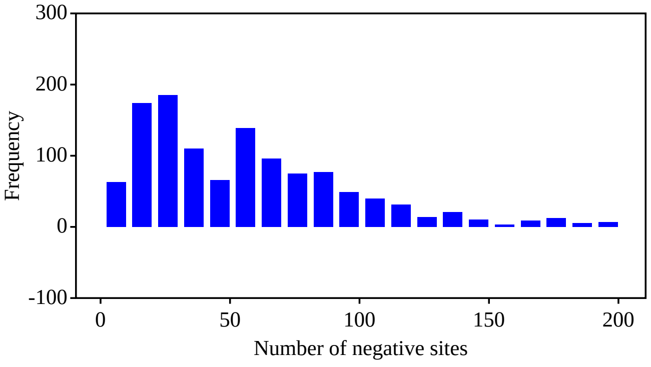

(b)

Figure 5: Distributions of the number of sites for each protein structure. The positive sites (a), and the negative sites (b). The x-axis indicates the number of detected sites for each query protein, whilst the y-axis indicates the frequency of proteins.

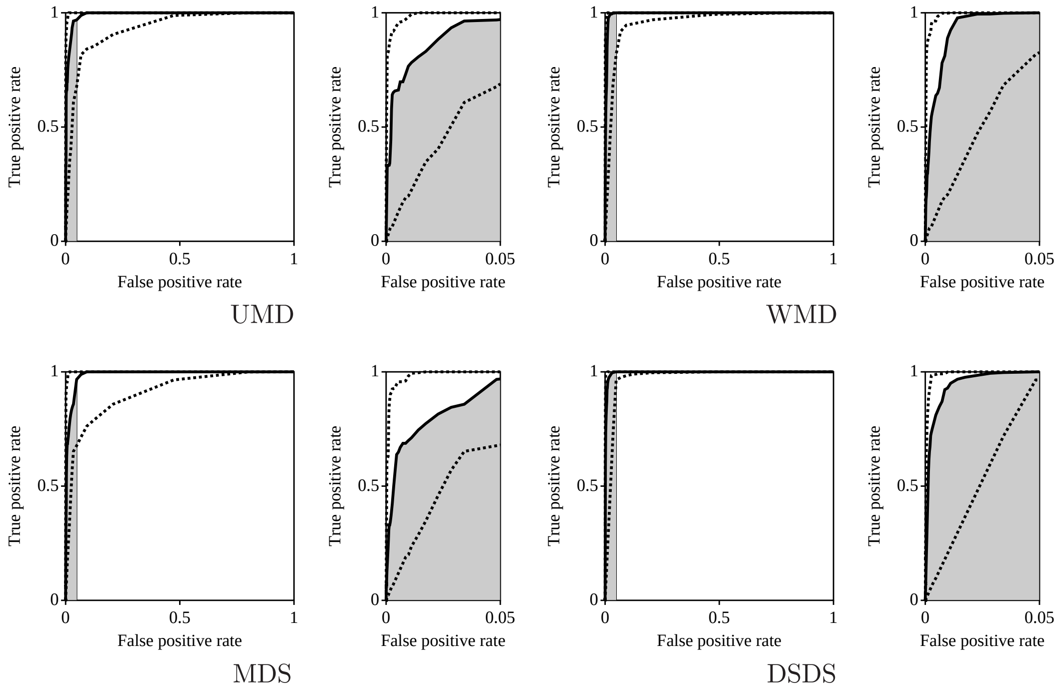

Figure 7: Median ROC curves for single template analysis. The ROC curve is computed for each method and each template. The solid line in each figure plots the median of the sensitivities over 36 templates at every specificity value. The dotted lines indicate the 25th percentile and the 75th percentile.

Table 4: An example of search results by the UMD measurement in the single template analysis

Rank	PDBid	Residues	RMSD
1	1g78	TYR 129 A, LYS 72 A	0.659
2	7yas	TYR 158 A, LYS 236 A	0.665
3	2yas	TYR 158 A, LYS 236 A	0.686
4	6yas	TYR 158 A, LYS 236 A	0.689
5	1g79	TYR 129 A, LYS 72 A	0.691
6	1dli	TYR 53 A, LYS 57 A	0.695
7	1lwd	TYR 68 A, LYS 41 A	0.726
8	1yas	TYR 158 A, LYS 236 A	0.736
9	1tec	TYR 149 E, LYS 153 E	0.740
10	1lwd	TYR 68 B, LYS 41 B	0.743

Template 1ams is used to generate this example.

Table 5: An example of search results by the UMD-PINTS measurement in the multiple template analysis

	Residues	Template	$\log(F_{\text{pints}})$	Deviation
1	ASP 137 A, ASP 194 A	2bvw	−8.77	0.82
2	ASP 153 A, HIS 71 A	1emh	−4.42	1.18
3	ASP 100 A, ASP 97 A	1qk2	−3.89	1.00
4	ASP 97 A, HIS 57 A	1emh	−2.38	1.27
5	ASP 189 A, HIS 172 A	1emh	−0.98	1.33
6	ASP 137 A, ASP 194 A	1qk2	1.07	1.23
7	ASP 61 A, ASP 61 A	1qk2	2.46	1.31
8	ASP 128 A, ASP 129 A	1psa	3.08	1.37
9	ASP 102 A, ASP 97 A	1qk2	3.64	1.37
10	ASP 189 A, HIS 146 A	1emh	3.81	1.56

Protein structure 1bio is used to generate this example.

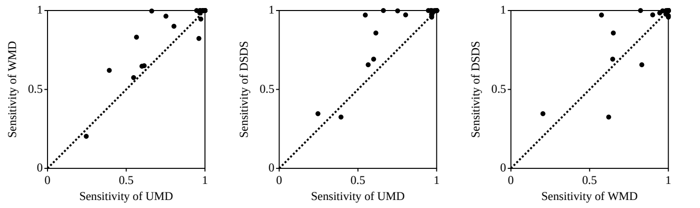

(a) (b) (c)

Figure 6: Scatter plots of Sensitivities in single template analysis. WMD vs UMD (a); DSDS vs UMD (b); DSDS vs WMD (c). The Sensitivities for 36 templates are plotted in each diagram.

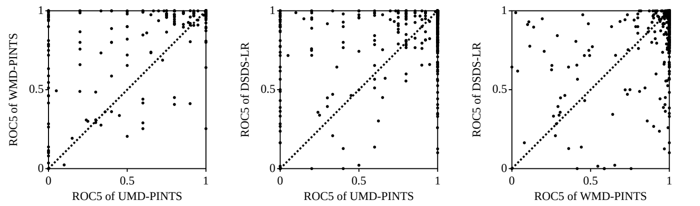

(a) (b) (c)

Figure 8: Scatter plots of ROC5 scores in multiple template analysis. WMD-PINTS vs UMD-PINTS (a); DSDS-LR vs UMD-PINTS (b); DSDS-LR vs WMD-PINTS (c). The scores for 1,219 protein structures are plotted in each diagram.

References

[1] A. Stark, S. Sunyaev, and R. B. Russell, "A model for statistical significance of local similarities in structure.," *J Mol Biol*, vol. 326, pp. 1307–16, Mar 2003.

[2] J. W. Torrance, G. J. Bartlett, C. T. Porter, and J. M. Thornton, "Using a library of structural templates to recognise catalytic sites and explore their evolution in homologous families.," *J Mol Biol*, vol. 347, pp. 565–81, Apr 2005.
